# Bactericidal, Bacteriostatic, and Fungicidal Activities of *Clausena anisata* Fruit and Its Compounds, Stigmasteryl 3‐Palmitate (a Phytosterol Ester) and Phellopterin (a Furanocoumarin), Against Some Clinical Pathogens

**DOI:** 10.1002/mbo3.70261

**Published:** 2026-03-01

**Authors:** Emmanuel Kofi Kumatia, Alex Asase, Prince Kyei Baffour, Borge Leth Frimpong, Ernest Agyei, Nguyen Huu Tung

**Affiliations:** ^1^ Department of Phytochemistry Centre for Plant Medicine Research Mampong‐Akuapem Ghana; ^2^ Plant Development Department Centre for Plant Medicine Research Mampong‐Akuapem Ghana; ^3^ Department of Microbiology Centre for Plant Medicine Research Mampong‐Akuapem Ghana; ^4^ Department of Medicinal Chemistry and Drug Quality Control, Faculty of Pharmacy Phenikaa University Hanoi Vietnam

**Keywords:** antibacterial activity, antifungal activity, antimicrobial activity, *Clausena anisata*, coumarin, minimum lethal concentration, phytosterol

## Abstract

The emergence of antimicrobial resistance necessitates the exploration of novel therapeutic agents from natural sources. This study investigated the antimicrobial properties of *Clausena anisata* fruit ethanol extract (CAFE) and its isolated compounds against 11 bacterial and three fungal strains. The dried fruits were extracted with ethanol using a Soxhlet apparatus. The extract was partitioned and subjected to chromatography to isolate two compounds, which were characterized as stigmasteryl 3‐palmitate (C‐01) and phellopterin (C‐02) using NMR spectroscopy and LC‐MS analysis. CAFÉ demonstrated notable inhibition zones in agar well diffusion assays, with the strongest activity against *Klebsiella pneumoniae* (14.67 ± 2.08 mm) and *Staphylococcus saprophyticus* (13.67 ± 0.58 mm). Microbroth dilution assays revealed MIC values ranging from 0.0781 to 1.2500 mg/mL for CAFÉ and 0.0781 to 1.2500 mg/mL for stigmasteryl 3‐palmitate and phellopterin, respectively. CAFE demonstrated bactericidal activity (MLC/MIC ≤ 4) against *Pseudomonas aeruginosa*, *Proteus mirabilis*, *Salmonella typhimurium*, *K. pneumoniae*, *Streptococcus sanguis*, *S. saprophyticus*, and *Candida glabrata*, while showing bacteriostatic activity (MLC/MIC > 4) against *Candida albicans*. Stigmasteryl 3‐palmitate exhibited bactericidal activity against *P. mirabilis*, *S. typhimurium*, *K. pneumoniae*, and *S. sanguis*, with bacteriostatic effects against the other organisms. Phellopterin demonstrated primarily bacteriostatic activity except against *S. saprophyticus*. Both compounds showed potent fungicidal activity against *Candida* species. These findings highlight the therapeutic potential of *C. anisata* fruit and its constituents against typhoid fever, hospital‐acquired pneumonia, and invasive candidiasis. This is the first report on the antibacterial and antifungal activities of *C. anisata* fruit, stigmasteryl 3‐palmitate, and phellopterin.

## Graphical abstract

*C. anisata* fruit extract and its compounds demonstrated potent broad‐spectrum antimicrobial activity, notably against *Klebsiella pneumoniae* (MIC 0.625 mg/mL; MLC 2.5 mg/mL) and *Candida albicans* (MIC 0.0781 mg/mL; MLC 0.625 mg/mL). The findings highlight promising bactericidal and fungicidal potential of stigmasteryl 3‐palmitate and phellopterin against clinically relevant pathogens.

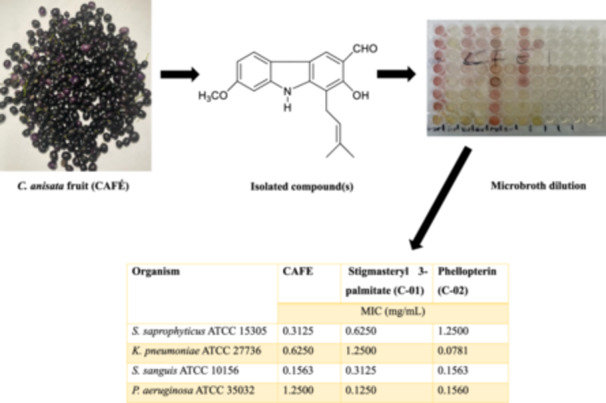

## 1 Introduction

The global burden of infectious diseases continues to escalate due to the widespread emergence of multidrug‐resistant (MDR) pathogens, creating an urgent need for novel antimicrobial agents (Cowan 1999). Indeed, the worldwide burden of antimicrobial resistance (AMR) constitutes a paramount threat to present‐day medical practice and public health frameworks. During the past 20 years (2000–2024), the evolution and transmission of these MDR organisms have intensified at an alarming pace, with a grave public health challenge (Centers for Disease Control and Prevention 2023). Early AMR concerns from the 1940s have evolved into a contemporary crisis, with resistant infections now causing over 1.27 million annual deaths and compromising healthcare systems worldwide (Murray et al. 2022; World Health Organization 2023). Traditional antibiotics are becoming increasingly ineffective against pathogenic bacteria and fungi, prompting researchers to explore alternative therapeutic approaches, particularly those derived from natural sources (Pandey and Kumar 2013). Medicinal plants have served as primary sources of therapeutic compounds for centuries, with approximately 70%–80% of rural populations in developing countries relying on plant‐based medicines for primary healthcare (World Health Organization 2019).

*Clausena anisata* (Willd.) Hook. f. ex Benth., commonly known as horsewood, belongs to the Rutaceae family and is widely distributed across sub‐Saharan Africa and tropical Asia (Omara et al. 2022). This deciduous shrub or small tree has been extensively utilized in traditional African medicine for treating various ailments, including bacterial and fungal infections, malaria, fever, and inflammatory conditions (Lawal et al. 2015). In Ghana, the plant is locally known as “Ayida” in Ewe and has been traditionally employed for treating skin infections, boils, ringworm, and eczema (Agyepong et al. 2014).

Phytochemical investigations of *C. anisata* have revealed the presence of diverse bioactive compounds, including alkaloids, coumarins, limonoids, phenylpropanoids, and essential oils (Chakraborty et al. 1995; Kumatia et al. 2017; Omara et al. 2022). Previous studies have demonstrated that various parts of the plant possess anti‐cancer, antimicrobial, analgesic, anti‐inflammatory, antioxidant, anti‐trypanosomal, and anti‐plasmodial activities (Chakraborty et al. 1995; Makirita et al. 2016; Kumatia et al. 2017; Kumatia et al. 2024). However, limited research has been conducted on the antimicrobial properties of *C. anisata* fruits, despite the traditional use of the plant in folk medicine.

Stigmasterol and its derivatives represent an important class of phytosterols with documented biological activities, including antimicrobial, anti‐inflammatory, and cholesterol‐lowering properties (Bakrim et al. 2022). Phytosterol esters have been reported to have enhanced bioavailability and efficacy compared to free phytosterols (Bakrim et al. 2022). Similarly, coumarins like phellopterin have demonstrated significant antimicrobial potential against various pathogens through multiple mechanisms of action (Walasek et al. 2015).

The present study aimed to investigate the antimicrobial activities of *C. anisata* fruit ethanol extract and characterize its bioactive constituents. Specifically, we sought to isolate and identify the major antimicrobial compounds, evaluate their individual antimicrobial efficacy, and determine their mechanisms of action against selected bacterial and fungal pathogens.

## 2 Materials and Methods

### 2.1 Chemicals and Other Materials

Ethanol (99%) was procured from Midland Ghana Limited, Tema. Ethyl acetate and petroleum ether (40°C–60°C) were purchased from Park Scientific Limited (Northampton, U.K.). Normal phase silica gel for CC (40–60 µm and 230 × 400 mesh size) and normal phase TLC plate (aluminum sheets coated silica gel 60F_254_) was supplied by Sorbent Technologies (Atlanta, GA., USA) and Merck Chemicals (Darmstadt, Germany), respectively.

### 2.2 Microorganisms

The *Salmonella typhi* strains CL1, CL2, and CL4 used in this study were archived clinical isolates previously obtained from the Clinical Microbiology Department, Korle Bu Teaching Hospital, Accra, Ghana, and preserved at (−80°C) at the Microbiology Department of the Centre for Plant Medicine Research (CPMR), Ghana. No patient samples were collected for this study. The non‐clinical micro‐organisms were purchased from a licensed laboratory.

### 2.3 Collection and Extraction of *C. anisata* Fruit

*C. anisata* was identified and authenticated at the premises of CPMR, Mampong‐Akuapem, Ghana, by Dr. Emmanuel Kofi Kumatia, the first author of this article, and a Pharmacognosist at the CPMR. The ripe fruit of the plant was collected in April 2023, after permission was obtained. The fruit was washed with clean water, sun‐dried for 10 days, and oven‐dried at 60°C for 48 h (Kumatia et al. 2024). Voucher specimen of the plant (CPMR 5101) was also deposited at the herbarium of CPMR, Mampong‐Akuapem, Ghana.

### 2.4 Extraction and Partitioning of *C. anisata* Fruit

The dried fruit (400 g) was pulverized into course powder and extracted with absolute ethanol (1 L × 3) for 6 h each in the Soxhlet apparatus. The resultant extract was concentrated to 240 mL using the rotary evaporator. Approximately 50 mL out of the 240 mL of the extract evaporated to dryness to obtain a brown solid, coded CAFE (7.2559 g) (Kumatia et al. 2024). The remaining 190 mL was diluted with distilled water to a final volume of 400 mL and partitioned with petroleum ether (4 × 400 mL). The aqueous layer was again extracted with Ethyl acetate (4 × 400 mL). Using the rotary evaporator, the Petroleum ether and Ethyl acetate fractions were dried to solids, which were coded CAFE‐P (5.0 g) and CAFE‐E (1.66 g), respectively.

### 2.5 Isolation of C‐01

Compound C‐01was isolated from the petroleum ether fraction of *C. anisata* fruit extract (CAF‐P) as follows. The CFE‐P (5.0 g) was absorbed into normal‐phase silica gel (10.0 g) and dried for 12 h. The dried mixture was loaded into a glass column packed with 170.0 g of normal‐phase silica gel. The elution of the column was started with Petroleum ether (200 mL), followed by the introduction of 5% Chloroform, then at an incremental rate of 10% Chloroform in order of increasing polarity until 100% Chloroform was attained. Ethanol was then introduced into the Chloroform at 10% rate until 100% Ethanol was also attained. The total volume of each solvent system used was 300 mL. A total of 78 fractions were eluted from the column in aliquots of about 200 mL each and grouped into eight major fractions (CAF‐P1 – CAF‐P8) based on their TLC profiles. C‐01 was obtained from CAF‐P4.

### 2.6 Isolation of Compounds from the Ethyl Acetate Fraction of *C. anisata* Fruit (CFE‐E)

CAF‐E (1.66 g) was absorbed into 4.0 g of normal phase silica gel and dried overnight. The sample was ten loaded into a column packed with 40 g of normal phase silica gel and eluted with Petroleum ether—Ethyl acetate—Ethanol system, starting with Petroleum ether and adding Ethyl acetate at 10% incremental rate. When 100% Ethyl acetate was attained, Ethanol was also added to the Ethyl acetate at 10% incremental rate until 100% Ethanol was attained. A total of 86 fractions were eluted from the column and grouped into eight (CAF‐E1–CAF‐E8) using their TLC chromatograms. C‐02 (112.30 mg) was obtained from fraction CAF‐E1.

## 3 Identification of Isolated Compounds

### 3.1 LC‐MS Analysis

The compounds were dissolved in acetonitrile. Aliquots of 2 mL per compound was analyzed using UHPL—MS/MS to determine the molecular mass of the compounds.

### 3.2 NMR Analysis of Compounds

Bruker FT‐NMR Spectrometer (Avance TM 500 MHz, Germany) coupled with Bruker\TopSpin3.6.3, JJ 10 NMR data analysis software was employed to generate the NMR data (^1^H, ^13^C, ^1^H‐^1^H COSY, DEPT135 and HMBC) of the compounds at room temperature. Tetramethylsilane was used as the internal standard and the chemical shift values given in *δ* (ppm). The compounds were dissolved in CDCl_3_ and the solutions used for the analysis (Kumatia et al. 2023).

## 4 Agar Well Diffusion Assay

The agar well diffusion test was performed according to the method described by Balouiri et al. (2016) with minor modifications. Mueller‐Hinton agar plates were prepared by autoclaving at 121°C for 15 min and pouring approximately 20–25 mL into sterile Petri dishes, then allowed to solidify at room temperature. Bacterial suspensions equivalent to 0.5 McFarland standard (approximately 1.5 × 10⁸ CFU/mL) were prepared in sterile saline and uniformly spread across the agar surface using sterile cotton swabs in two perpendicular directions, followed by a 3–5 min drying period. Wells of 6–8 mm diameter were cut into the inoculated agar using a sterile cork borer, with agar plugs removed using sterile forceps, ensuring wells were evenly spaced and at least 15 mm from plate edges. Test solutions (50–100 μL) were dispensed into wells using micropipettes, along with appropriate positive controls (standard antibiotics) and negative controls (solvent only), then allowed to diffuse for 30 min at room temperature before incubation at 37°C for 18–24 h. Antimicrobial activity was evaluated by measuring the diameter of clear inhibition zones around wells using calipers, with results recorded in millimeters and compared against controls to determine the relative antimicrobial potency of test agents (Valgas et al. 2007).

## 5 Minimum Inhibitory Concentration (MIC) and Minimum Lethal Concentration (MLC)

The MIC is to show the least concentration of the extract that can have inhibitory activity against the isolates. In this study, the MICs were deternimed by employing the microbroth dilution assay described by Agyepong et al. (2014) with some modifications. The organisms were reconstituted into nutrient broth at 1.0 × 10^9^ CFU/mL. Approximately, 10 mg/mL of each extract was aliquoted in the first well containing 100 µL of double‐strength nutrient broth and serially diluted along the wells. Ciprofloxacin (15 uL) or Miconazole (150 uL) was used as positive control for bacterial and fungi strains respectively. Plates were incubated at 32°C for 24 h after the organisms were added to each respective well. After 24 h, a loopful of inoculum from each well was plated on Nutrient Agar and incubated at 32°C. About 40 µL of iodonitrotetrazolium chloride (INT) dye was added to each well and incubated for at 32°C for 45 min.

### 5.1 Indications

#### 5.1.1 MIC

Wells with red‐pink coloration/precipitation were indicative of no inhibitory microbial reaction. The results were recorded and analyzed.

#### 5.1.2 MLC

Wells with inoculum showing growth on Nutrient Agar is indicative of the presence of organisms, whereas wells with inoculum showing no growth is indicative of absence of microbial growth. The well with the least concentration that showed activity is designated as the MLC.

## 6 Results

### 6.1 Identification of the Compounds Isolated From *Clausena* Fruit Extract (CAFE)

**Figure 1:**
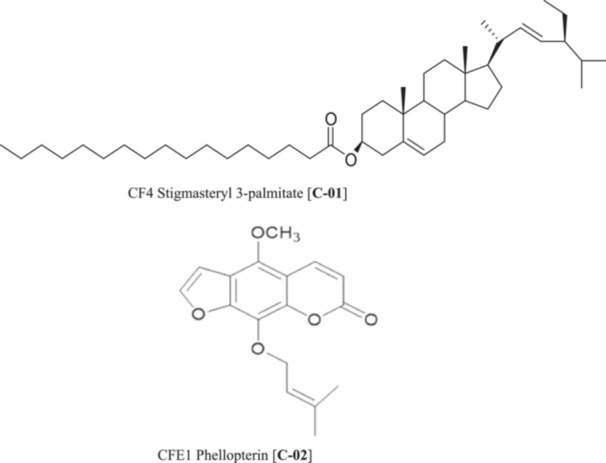
Chemical structures of compounds isolated from *C. anisata* fruit extract.

The compounds isolated from CAFE were characterized as Stigmasteryl 3‐palmitate [**C‐01**] and Phellopterin [**C‐02**] based on their NMR and LC‐MS data (Figure 1).

#### 6.1.1 Identification of **C‐01** as Stigmasteryl 3‐palmitate

**C‐01** was obtained as a colorless oil. ^1^H NMR (CDCl_3_, 500 MHz): stigmasteryl moiety: *δ* 5.34 (1H, brd, *J* = 6.0 Hz, H‐6), 5.15 (1H, brd, *J* = 6.5, H‐22), 5.05 (1H, brd, *J* = 6.5 Hz, H‐23), 3.52 (1H, m, H‐3), 1.06 (3H, s, H‐19), 1.01 (3H, d, *J* = 6.5 Hz, H‐21), 0.83 (3H, d, *J* = 6.0 Hz, H‐26), 0.80 (3H, *J* = 6.0 Hz, H‐27), 0.66 (3H, s, H‐18), 0.64 (3H, t, *J* = 6.5 Hz, H‐29), palmitoyl moiety: *δ* 2.38 (2H, m, H‐2ʹ), 1.70–1.80 (2H, m, H‐3ʹ), 1.10‐1.40 (26H, H‐4ʹ—H‐16ʹ), 0.85 (3H, t, *J* = 6.5 Hz, H‐16ʹ); ^13^C NMR (CDCl_3_, 125 MHz): stigmasteryl moiety: *δ* 37.2 (C‐1), 31.6 (C‐2), 72.8 (C‐3), 42.2 (C‐4), 140.7 (C‐5), 121.7 (C‐6), 32.0 (C‐7), 31.9 (C‐8), 50.2 (C‐9), 36.5 (C‐10), 21.1 (C‐11), 39.8 (C‐12), 42.3 (C‐13), 56.7 (C‐14), 24.6 (C‐15), 28.7 (C‐16), 56.0 (C‐17), 11.9 (C‐18), 19.3 (C‐19), 40.5 (C‐20), 21.5 (C‐21), 138.4 (C‐22), 129.3 (C‐23), 51.2 (C‐24), 36.1 (C‐25), 19.8 (C‐26), 18.9 (C‐27), 25.3 (C‐28), 12.0 (C‐29), palmitoyl moiety: *δ* 174.2 (C‐1ʹ), 35.1 (C‐2ʹ), 29.0–31.0 (C‐3ʹ‐C‐14ʹ), 24.5 (C‐15ʹ), 14.1 (C‐16ʹ). ESI‐MS (positive): *m/z* 651.7 [M + H]^+^, 256.3 [C_16_H_32_O_2_]^+^, 413.2 [C_29_H_49_O]^+^.

#### 6.1.2 Identification of **C‐02** as Phellopterin

**C‐02**: was recovered as a white amorphous powder; ^1^H NMR (CD_3_OD, 500 MHz): *δ* 8.10 (1H, d, *J* = 9.5 Hz, H‐4), 7.80 (1H, d, *J* = 2.5 Hz, H‐2ʹ), 7.18 (1H, d, *J* = 2.5 Hz, H‐3ʹ), 6.25 (1H, d, *J* = 9.5 Hz, H‐3), 5.52 (1H, t, *J* = 6.5, H‐2"), 4.88 (1H, d, *J* = 6.5, H‐1"), 4.20 (3H, s, 5‐OCH_3_), 1.71 (3H, s, H‐4"), 1.65 (3H, s, H‐5"); ^13^C NMR (CD_3_OD, 125 MHz): *δ* 161.4 (C‐2), 150.9 (C‐7), 145.5 (C‐2ʹ), 145.6 (C‐3"), 144.6 (C‐8a), 144.0 (C‐5), 140.0 (C‐4), 139.4 (C‐8), 119.5 (C‐2"), 114.5 (C‐6), 111.5 (C‐3), 106.9 (C‐4a), 105.0 (C‐3ʹ), 69.6 (C‐1"), 59.9 (5‐OCH_3_), 24.5 (C‐4"), 16.6 (C‐5"); ESI‐MS (positive): *m/z* 301.3 [M + H]^+^ (Nakatani 1991).

### 6.2 Antimicrobial Activity Results ‐ Zone of Inhibition From Agar Well Diffusion Assay

The agar well diffusion assay demonstrated variable zones of inhibition for CAFE against the tested microorganisms (Table 1). Among bacterial pathogens, the largest inhibition zones were observed against *K. pneumoniae* ATCC 27736 (14.67 ± 2.08 mm) and *S. saprophyticus* ATCC 15305 (13.67 ± 0.58 mm), followed by *S. typhi* CL1 (12.67 ± 1.15 mm).

**Table 1:** Results of zone of inhibition (mm) expressed as mean (standard deviation).

Organism	CAFE	Negative control (DSMO)	Standard drugs	
			Ciprofloxacin	Miconazole
*S. typhi* NTCC 33433	6.00 (0.00)	6.00 (0.00)	34.33 (0.58)	—
*K. pneumoniae* ATCC 27736	14.67 (2.08)	6.00 (0.00)	26.00 (1.00)	—
*S. aureus*	6.00 (0.00)	6.00 (0.00)	32.33 (1.15)	—
*N. gonorrhea*	9.00 (1.00)	6.00 (0.00)	28.33 (0.58)	—
*S. typhimirium* ATCC 14029	11.00 (1.00)	6.00 (0.00)	34.00 (1.00)	—
*E. coli* ATCC	11.00 (0.00)	6.00 (0.00)	30.00 (0.00)	—
*S. typhi* CL1	12.67 (1.15)	6.00 (0.00)	28.33 (1.15)	—
*S. typhi* CL4	6.00 (0.00)	6.00 (0.00)	30.67 (0.58)	—
*S. saprophyticus* ATCC 15305	13.67 (0.58)	6.00 (0.00)	17.67 (0.58)	—
*S. typhi* CL2	10.00 (0.00)	6.00 (0.00)	33.67 (0.58)	—
*P. mirabilis* ATCC 5659	10.00 (1.00)	6.00 (0.00)	39.33 (0.58)	—
*C. albicans* ATCC 10231	9.33 (0.58)	6.00 (0.00)	—	6.00 (0.00)
*E. flucossum* ATCC 52066	15.67 (3.06)	6.00 (0.00)	—	15.00 (1.00)
*C. glabrata*	11.33 (1.53)	6.00 (0.00)	—	6.00 (0.00)

*Note:* CAFE = *C. anisata* fruit ethanol extract, DMSO (dimethyl sulfoxide); Ciprofloxacin = Positive control for bacteria; Miconazole = Positive control for fungi; “—” indicates not applicable; bacteria are listed first (rows 1–11), followed by fungi (rows 12–14).

Moderate inhibition zones ranging from 9.00 to 11.00 mm were recorded for *S. typhimirium* ATCC 14029 (11.00 (1.00) mm), *E. coli* ATCC (11.00 (0.00) mm), *S. typhi* CL2 (10.00 (0.00) mm), *P. mirabilis* ATCC 5659 (10.00 (1.00) mm), and *N. gonorrhea* (9.00 (1.00) mm). No inhibitory activity was detected against *S. typhi* NTCC 33433, *S. aureus*, and *S. typhi* CL4, all showing zones identical to the negative control (6.00 (0.00) mm). For fungal pathogens, *E. flucossum* ATCC 52066 exhibited the largest inhibition zone (15.67 (3.06) mm), while *C. glabrata* and *C. albicans* ATCC 10231 showed zones of 11.33 (1.53) mm and 9.33 (0.58) mm, respectively. Standard drugs ciprofloxacin and miconazole consistently produced larger inhibition zones than CAFE across all susceptible organisms.

### 6.3 MIC

**Figure 2:**
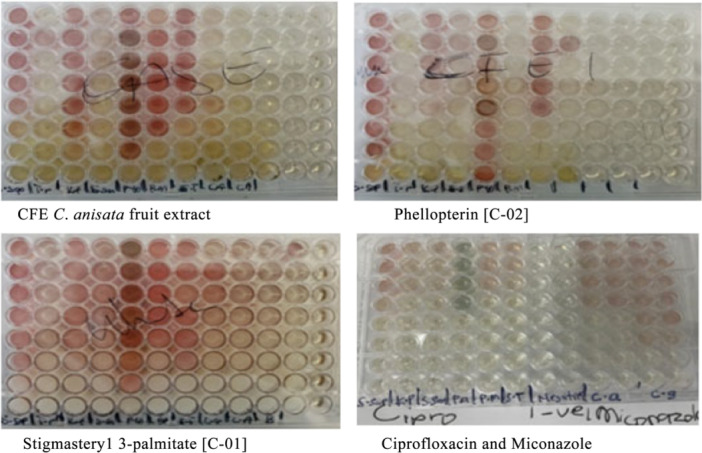
Images of the 96‐well plates showing colorimetric results of the microbroth dilution assay after 24 h of incubation of the bacteria and fungi with the drugs and extracts. The negative controls are the last two rows on the right side of each plate or the middle for the standard drugs.

The colorimetric outcomes of the microbroth dilution assay using iodonitrotetrazolium chloride (INT) and the MICs obtained are presented below in Figure 2 and Table 2, respectively. The INT reagent was initially colorless in the nutrient medium. However, after incubation with the test microorganisms and plant extracts or standard antimicrobial drugs, some of the wells turned pink, or light yellow to nearly colorless solutions. The negative control (nutrients only) wells remained colorless even after incubation.

**Table 2:** Minimum inhibitory concentration (MIC) results (mg/mL).

Organism	CAFE	Stigmasteryl 3‐palmitate (C‐01)	Phellopterin (C‐02)	Ciproflo‐xacin	Miconazole
*S. saprophyticus* ATCC 15305	0.3125	0.6250	1.2500	0.00375	—
*K. pneumoniae* ATCC 27736	0.6250	1.2500	0.0781	0.000234375	—
*S. sanguis* ATCC 10156	0.1563	0.3125	0.1563	0.000234375	—
*P. aeruginosa* ATCC 35032	1.2500	0.1250	0.1560	0.00375	—
*P. mirabilis* ATCC 5659	0.6250	0.3125	0.0781	0.001875	—
*S. typhimirium* ATCC 14029	0.6250	0.6250	0.3125	0.000234375	—
*C. glabrata* ATCC MYA 2950	0.6250	0.0781	0.0781	—	0.0375
*C. albicans* ATCC 10231	0.0781	0.0781	0.0781	—	0.009375

Furthermore, the MIC values varied considerably among the test compounds and target microorganisms (Table 2). CAFE demonstrated MIC values ranging from 0.0781 mg/mL against *C. albicans* to 1.2500 mg/mL against *P. aeruginosa*, with intermediate values of 0.1563 mg/mL for *S. sanguis*, 0.3125 mg/mL for *S. saprophyticus*, and 0.6250 mg/mL for *K. pneumoniae*, *P. mirabilis*, *S. typhimirium*, and *C. glabrata*.

Stigmasteryl 3‐palmitate exhibited MIC values from 0.0781 mg/mL against both *Candida* species to 1.2500 mg/mL against *K. pneumoniae*, with notable activity at 0.1250 mg/mL against *P. aeruginosa* and 0.3125 mg/mL against *P. mirabilis* and *S. sanguis*. Phellopterin displayed MIC values ranging from 0.0781 mg/mL against *C. glabrata*, *P. mirabilis*, and both *Candida* species to 1.2500 mg/mL against *S. saprophyticus*, showing intermediate values of 0.1563 mg/mL for *S. sanguis* and *P. aeruginosa*, and 0.3125 mg/mL for *S. typhimirium*. The reference antibiotics ciprofloxacin and miconazole exhibited substantially lower MIC values, with ciprofloxacin ranging from 0.000234375 mg/mL to 0.00375 mg/mL and miconazole from 0.009375 mg/mL to 0.0375 mg/mL.

### 6.4 MLC

The MLC determinations revealed higher concentration requirements compared to MIC values across all test substances (Table 3). CAFE showed MLC values ranging from 0.6250 mg/mL for *S. sanguis* and *C. albicans* to 2.5000 mg/mL for *K. pneumoniae* and *P. aeruginosa*, with intermediate values of 1.2500 mg/mL recorded for *S. saprophyticus*, *P. mirabilis*, *S. typhimirium*, and *C. glabrata*.

**Table 3:** Minimum lethal concentration (MLC) results (mg/mL).

Organism	CAFE	Stigmasteryl 3‐palmitate (C01)	Phellopterin (C‐02)	Ciproflo‐xacin	Miconazole
*S. saprophyticus* ATCC 15305	1.2500	5.0000	5.0000	0.00375	—
*K. pneumoniae* ATCC 27736	2.5000	2.5000	2.5000	0.00046875	—
*S. sanguis* ATCC 10156	0.6250	0.6250	1.2500	0.00046875	—
*P. aeruginosa* ATCC 35032	2.5000	2.5000	5.0000	0.00375	—
*P. mirabilis* ATCC 5659	1.2500	0.3125	1.2500	0.00375	—
*S. typhimirium* ATCC 14029	1.2500	0.6250	2.5000	0.00046875	—
*C. glabrata* ATCC MYA 2950	1.2500	0.6250	2.5000	—	0.075
*C. albicans* ATCC 10231	0.6250	0.6250	2.5000	—	0.01875

Stigmasteryl 3‐palmitate exhibited MLC values from 0.3125 mg/mL against *P. mirabilis* to 5.0000 mg/mL against *S. saprophyticus* and *P. aeruginosa*, with values of 0.6250 mg/mL for both *Candida* species and *S. sanguis*, and 2.5000 mg/mL for *K. pneumoniae*. Phellopterin demonstrated MLC values ranging from 1.2500 mg/mL for *S. sanguis* and *P. mirabilis* to 5.0000 mg/mL for *S. saprophyticus* and *P. aeruginosa*, with 2.5000 mg/mL recorded for *K. pneumoniae*, *S. typhimirium*, and *C. glabrata*, and *C. albicans*. The standard drugs maintained their superior potency with ciprofloxacin MLC values from 0.00046875 to 0.00375 mg/mL and miconazole from 0.01875 to 0.075 mg/mL.

### 6.5 MLC to MIC Ratios

The calculated MLC/MIC ratios (Table 4) revealed distinct patterns of antimicrobial action among the tested compounds. CAFE produced ratios ranging from 2.00 against *P. aeruginosa*, *P. mirabilis*, and *S. typhimirium* to 8.00 against *C. albicans*, with ratios of 4.00 recorded for *S. saprophyticus*, *K. pneumoniae*, *S. sanguis*, and *C. glabrata*.

**Table 4:** MLC/MIC ratios for CAF, compounds, and standard drugs.

Organisms	CAFE	Stigmasteryl 3‐palmitate (C‐01)	Phellopterin (C‐02)	Ciprofloxacin	Miconazole
*S. saprophyticus* ATCC 15305	4.00	8.00	4.00	1.00	—
*K. pneumoniae* ATCC 27736	4.00	2.00	32.01	2.00	—
*S. sanguis* ATCC 10156	4.00	2.00	8.00	2.00	—
*P. aeruginosa* ATCC 35032	2.00	20.00	32.05	1.00	—
*P. mirabilis* ATCC 5659	2.00	1.00	16.01	2.00	—
*S. typhimirium* ATCC 14029	2.00	1.00	8.00	2.00	—
*C. glabrata* ATCC MYA 2950	2.00	8.00	32.01	—	2.00
*C. albicans* ATCC 10231	8.00	8.00	32.01	—	2.00

Stigmasteryl 3‐palmitate showed ratios from 1.00 for *P. mirabilis* and *S. typhimirium* to 20.00 for *P. aeruginosa*, with intermediate values of 2.00 for *K. pneumoniae* and *S. sanguis*, and 8.00 for *S. saprophyticus*, *C. glabrata*, and *C. albicans*. Phellopterin exhibited ratios ranging from 4.00 against *S. saprophyticus* to 32.05 for *K. pneumoniae*, *P. aeruginosa*, *C. glabrata*, and *C. albicans*, with intermediate values of 8.00 for *S. sanguis* and *S. typhimirium*, and 16.01 for *P. mirabilis*. The reference drugs ciprofloxacin and miconazole consistently maintained low ratios of 1.00 to 2.00 across all tested organisms, indicating consistent antimicrobial performance.

## 7 Discussion

This study represents the first comprehensive investigation of the antimicrobial properties of *C. anisata* fruit and its isolated bioactive constituents. The results demonstrate significant antimicrobial potential against clinically important pathogens, supporting the traditional use of this plant in treating infectious diseases across Africa and Asia.

The agar well diffusion assay was employed as a preliminary screening tool to evaluate CAFE's antimicrobial potential due to its simplicity, cost‐effectiveness, and ability to test multiple organisms simultaneously (Balouiri et al. 2016). This method relies on antimicrobial agent diffusion through agar to create inhibition zones, providing semi‐quantitative assessment of activity (Valgas et al. 2007).

CAFE demonstrated broad‐spectrum antifungal activity, exhibiting measurable inhibition against *C. glabrata*, *C. albicans*, and *E. floccosum* in the agar well diffusion assay. Notably, the extract produced a zone of inhibition of 15.67 (3.06) mm against *E. floccosum*, which was comparable to the standard antifungal drug miconazole (15 (0.00) mm). Against *C. glabrata* (11.33 (1.53) mm) and *C. albicans* ATCC 10231 (9.33 (0.58) mm), CAFE showed clear inhibitory activity, whereas miconazole was inactive against these two species under the same experimental conditions. These findings indicate that the *C. anisata* fruit extract contains antifungal constituents with activity extending beyond the azole spectrum, possibly acting through alternative mechanisms. The pronounced efficacy against *C. glabrata*, a species frequently reported to exhibit intrinsic or acquired resistance to azole antifungal agents (Pfaller and Diekema 2007), further highlights the therapeutic potential of CAFE as a natural antifungal agent and supports its ethnomedicinal use in the management of fungal infections.

The isolated pure compounds (C‐01 and C‐02) were not evaluated using this method due to insufficient quantities obtained from tcolumn chromatography. Instead, the microbroth dilution MIC method was prioritized as it requires significantly smaller sample volumes (10–20 µL per well) compared to agar well diffusion (50–100 µL per well) and provides precise quantitative data on minimum inhibitory concentrations essential for determining antimicrobial potency (Wiegand et al. 2008). Following positive screening results, more detailed MIC and MLC determinations were conducted.

The INT reagent used in the microbroth dilution assay is usually colorless in the nutrient medium in the absence of microorganisms. However, in the presence of live micro‐organisms, the INT is reduced to formazan, which is pink, by the metabolic action of the organisms. When the extract or drug inhibits the growth of the organism, the wells show the color of the extract or drug instead of the pink color. The various colorations (pink, light yellow, or nearly colorless solutions) observed for the tested agents clearly demonstrate the differential antimicrobial effects of the tested extracts and drugs compared with the control groups. (Eloff 1998; Balouiri et al. 2016).

Based on established interpretive criteria, crude plant extracts with MIC values below 0.1 mg/mL are considered very active, those between 0.1 and 0.5 mg/mL as active, 0.5–1.0 mg/mL as moderately active, and above 1.0 mg/mL as inactive (Ríos and Recio 2005; Fabry et al. 1998; Kuete 2010). Pure compounds are generally classified as very active when MIC ≤ 1 µg/mL, active at 1–10 µg/mL, moderately active at 10–100 µg/mL, and inactive when MIC > 100 µg/mL (Kuete 2010). In this study, the ethanol extract of *C. anisata* fruit (CAFE) demonstrated MIC values ranging from 0.0781 to 1.2500 mg/mL, indicating active to moderately active antimicrobial potency against most of the tested microorganisms. The strongest inhibition was observed against *C.albicans* (MIC = 0.0781 mg/mL) and *S sanguis* (MIC = 0.1563 mg/mL), confirming notable antifungal and antibacterial efficacy. The isolated compounds, stigmasteryl 3‐palmitate and phellopterin, also exhibited significant activity, with MIC values between 0.0781 and 1.2500 mg/mL. Notably, phellopterin displayed the highest potency against *K. pneumoniae*, *P. mirabilis*, and *Candida* species (MIC = 0.0781 mg/mL), corresponding to a very active classification for a crude natural compound. These findings underscore that both the extract and its constituents possess promising broad‐spectrum antimicrobial properties.

The predominantly bacteriostatic nature of these compounds may be advantageous in clinical applications as it can reduce the likelihood of developing resistance while still providing therapeutic efficacy, though bactericidal activity is generally preferred for treating serious infections in immunocompromised patients (Pankey and Sabath 2004).

The analysis of MLC/MIC ratios provides crucial insights into the mechanism of action of CAFE and its isolated compounds. According to established criteria, antimicrobial agents are considered bactericidal when the MLC (or MBC) to MIC ratio is ≤ 4, while ratios > 4 indicate primarily bacteriostatic activity (French et al. 2006; Rodríguez‐Melcón et al. 2022). This classification system has been widely validated and is considered a fundamental principle in antimicrobial pharmacodynamics (Levison and Levison 2009).

Analysis of Table 4 reveals that most compounds demonstrated bacteriostatic activity against the majority of tested organisms. For CAFE, the MLC/MIC ratios ranged from 2.00 to 8.00, with ratios ≤ 4 observed against *P. aeruginosa* (2.00), *P. mirabilis* (2.00), *S. typhimirium* (2.00), *K. pneumoniae* (4.00), *S. sanguis* (4.00), *S. saprophyticus* (4.00), and *C. glabrata* (2.00), indicating bactericidal activity against these pathogens. However, ratios > 4 against *C. albicans* (8.00) suggest bacteriostatic action. Stigmasteryl 3‐palmitate showed bactericidal activity (ratios ≤ 4) against *P. mirabilis* (1.00), *S. typhimirium* (1.00), *K. pneumoniae* (2.00), and *S. sanguis* (2.00), but demonstrated bacteriostatic effects against other organisms with ratios ranging from 8.00 to 20.00. Notably, phellopterin exhibited predominantly bacteriostatic activity with most ratios > 4, except for *S. saprophyticus* (ratio = 4.00), which represents the threshold between bactericidal and bacteriostatic activity.

Among the organisms tested, several clinical isolates were included, notably *S. typhi* strains CL1, CL2, and CL4, which represent clinical isolates from patients with typhoid fever. Typhoid fever is a life‐threatening systemic infection caused by *S. enterica* serotype typhi. According to the 2019 Global Burden of Disease estimates, typhoid fever affects approximately 9 million people annually, resulting in about 110,000 deaths per year (World Health Organization 2023). The disease presents with high fever, headache, abdominal pain, and can lead to serious complications, including intestinal hemorrhage, bowel perforation, and death if left untreated. The demonstrated activity of CAFE and its isolated compounds against these clinical isolates (MIC values of 0.0781‐1.2500 mg/mL) suggests potential therapeutic applications for treating typhoid fever, particularly in regions where extensively drug‐resistant (XDR) strains are emerging (Klemm et al. 2018).

*K. pneumoniae* ATCC 27736, another key pathogen in this study, is an opportunistic gram‐negative, encapsulated enterobacterium that naturally inhabits the human oropharynx and gastrointestinal tract but poses serious risks in immunocompromised individuals and those with underlying conditions such as alcoholism or diabetes (Podschun and Ullmann 1998; Ashurst and Dawson 2023). This bacterium is the leading cause of nosocomial pneumonia in the United States, responsible for 3%–8% of all hospital‐acquired infections (Paczosa and Mecsas 2016). Its pathogenicity stems from multiple virulence factors such as a polysaccharide capsule that prevents immune recognition, lipopolysaccharides that trigger inflammatory responses, fimbriae that enable cellular adhesion, and siderophores that facilitate iron acquisition (Paczosa and Mecsas 2016). Alarmingly, *K. pneumoniae* has developed extensive antimicrobial resistance, with carbapenem resistance now characterizing 80% of carbapenem‐resistant Enterobacteriaceae infections (Centers for D. Control and P. 2013). Mortality remains exceptionally high, reaching 50%–100% in patients with alcoholism and bacteremia (Ashurst and Dawson 2023), emphasizing the critical need for alternative therapeutic strategies.

The notable activity observed against *K. pneumoniae* demonstrates promising therapeutic potential for treating these serious nosocomial infections, particularly given the global emergence of hypervirulent and carbapenem‐resistant strains. Phellopterin exhibited the strongest activity (MIC = 0.0781 mg/mL), followed by the crude ethanol extract CAFE (MIC = 0.6250 mg/mL) and stigmasteryl 3‐palmitate (MIC = 1.2500 mg/mL), suggesting that all three substances possess clinically relevant activity against this important pathogen.

The tested *Candida* species, *C. albicans* ATCC 10231 and *C. glabrata*, are major fungal pathogens causing significant morbidity and mortality (Kullberg and Arendrup 2015). *C. albicans* is the most common cause of candidemia globally, while *C. glabrata* has become the second most common causative agent in many geographical regions (Pfaller and Diekema 2007). These organisms cause infections ranging from superficial candidiasis to life‐threatening invasive candidiasis and bloodstream infections, particularly in immunocompromised patients. The superior antifungal activity of both isolated compounds against these *Candida* species (MIC values of 0.0781 mg/mL) is particularly noteworthy given the increasing prevalence of antifungal resistance and limited treatment options available.

The isolation and characterization of Stigmasteryl 3‐palmitate and phellopterin from *C. anisata* fruits adds to the growing body of knowledge regarding the phytochemical diversity of Rutaceae plants. Stigmasteryl 3‐palmitate, a phytosterol ester, represents an interesting class of bioactive compounds with enhanced solubility and bioavailability compared to free phytosterols (Wang et al. 2022). The esterification of stigmasterol with palmitic acid likely contributes to improved membrane penetration and antimicrobial efficacy, as previously reported for other phytosterol esters (Pereira et al. 2022).

Phytosterol esters demonstrated significantly enhanced oil solubility and bioavailability compared to their free forms, which is crucial for their therapeutic applications (Wang et al. 2024). The improved physicochemical properties of phytosterol esters result from the chemical modification that increases lipophilicity and facilitates cellular uptake through enhanced membrane interaction. Recent studies have shown that phytosterols play key roles in plant innate immunity against bacterial pathogens by regulating nutrient efflux and membrane integrity (Wang et al. 2012). Phellopterin, a furanocoumarin commonly found in Apiaceae and Rutaceae families, has been associated with various biological activities, including antimicrobial, anti‐inflammatory, and antioxidant properties (Kwiatkowski et al. 2015). The methoxy substitution in phellopterin may enhance its lipophilicity, facilitating penetration through microbial cell membranes and contributing to its antimicrobial activity. Coumarins have been shown to exhibit antimicrobial activity through various mechanisms including membrane disruption, DNA gyrase inhibition, and interference with DNA replication and protein synthesis (Cheke et al. 2022).

Recent comprehensive reviews have highlighted the diverse antimicrobial mechanisms of coumarin derivatives, including their ability to function as DNA gyrase inhibitors, which is particularly relevant for their antibacterial activity (Matos et al. 2015). Furanocoumarins like phellopterin have been shown to exhibit potent antimicrobial activity with MIC values as low as 0.40–0.46 μM against various pathogens, often surpassing conventional antibiotics in potency (Sardari et al. 1999; Celeghini et al. 2001). The structure‐activity relationship studies indicate that the furanocoumarin scaffold provides optimal antimicrobial activity, with the furan ring fusion and methoxy substitution patterns being crucial for biological activity (Appendino et al. 2007).

The broad‐spectrum antimicrobial activity observed for CAFE suggests a synergistic effect between multiple bioactive compounds present in the extract. This phenomenon is commonly observed in plant extracts where individual compounds work together to enhance overall antimicrobial efficacy, with recent studies emphasizing the importance of natural products in combating antimicrobial resistance (Wagner and Ulrich‐Merzenich 2009). The current global landscape emphasizes the urgent need for new antimicrobial agents derived from natural sources, with plant extracts representing a valuable reservoir of bioactive compounds with diverse mechanisms of action (Atanasov et al. 2021).

Several mechanisms may account for the observed antimicrobial activities. Phytosterols like stigmasterol can disrupt microbial cell membrane integrity by altering membrane fluidity and permeability, a mechanism that has been extensively studied using advanced microscopy techniques (Denkova et al. 2016). Recent cryo‐electron tomography studies have provided detailed visualization of membrane disruption mechanisms by antimicrobial compounds, showing that sterol‐like molecules can create pores and destabilize bacterial membranes. Coumarins such as phellopterin may interfere with DNA replication, protein synthesis, or enzyme function in target microorganisms through multiple pathways (Al‐Majedy et al. 2016). Recent studies have demonstrated that coumarin derivatives can function as potent DNA gyrase inhibitors, with some derivatives showing IC_50_ values comparable to standard antibiotics like novobiocin. The ability of coumarins to inhibit biofilm formation has also been extensively documented, representing an additional therapeutic advantage.

From a pharmaceutical development perspective, the identified compounds represent promising lead molecules for antimicrobial drug development. However, further optimization through structural modifications, formulation development, and extensive safety evaluation would be necessary before clinical applications. The development of novel drug delivery systems, including nanoencapsulation and targeted delivery approaches, could enhance the therapeutic potential of these natural compounds.

The study has some limitations, including the need for more comprehensive mechanistic studies, evaluation of synergistic effects between compounds, and assessment of cytotoxicity and safety profiles. Additionally, testing against a broader panel of clinically relevant pathogens, including MDR strains such as MRSA, VRE, and XDR bacteria, would strengthen the findings. Future studies should also include time‐kill kinetics, resistance development studies, and in vivo efficacy evaluations using appropriate animal models.

The investigation of potential synergistic interactions between the isolated compounds and conventional antibiotics represents a promising avenue for future research, as combination therapy has shown success in overcoming resistance mechanisms. Furthermore, detailed pharmacokinetic and pharmacodynamic studies would be essential to determine optimal dosing regimens and therapeutic windows.

## 8 Conclusion

This investigation demonstrated the antimicrobial potential of *C*. *anisata* fruit, identifying stigmasteryl 3‐palmitate and phellopterin as major bioactive constituents. Both compounds exhibited notable activity against bacterial and fungal pathogens, particularly Salmonella strains, *K. pneumoniae*, and *Candida species*. The MLC/MIC ratio analysis revealed predominantly bacteriostatic mechanisms, which may reduce the development of resistance while maintaining therapeutic efficacy. These findings validate the traditional medicinal use of *C. anisata* for treating infectious diseases. The broad‐spectrum activity and bacteriostatic mechanism indicate promising therapeutic potential in addressing antimicrobial resistance. Sustainable utilization of *C. anisata* fruit could contribute to pharmaceutical developments, supporting the growing demand for natural therapeutic alternatives.

## Author Contributions

Emmanuel Kofi Kumatia and Alex Asase conceived the research idea. Emmanuel Kofi Kumatia developed the experimental methods, performed the phytochemical studies and compound isolations, and authored the manuscript. Prince Kyei Baffour was also involved in the isolation of the compounds. Borge Leth Frimpong and Ernest Adjei performed the microbiological studies. Nguyen Huu Tung carried out the structure elucidation of the compounds and interpretation of the NMR data.

## Funding

The authors received no specific funding for this work.

## Ethics Statement

The authors have nothing to report.

## Conflicts of Interest

The authors declare no conflicts of interest.

## Data Availability

Data available on request from the authors.
